# Posterior internal fixation plus vertebral bone implantation under navigational aid for thoracolumbar fracture treatment

**DOI:** 10.3892/etm.2013.1087

**Published:** 2013-04-29

**Authors:** WEI ZHOU, WEIQING KONG, BIZHEN ZHAO, YISHAN FU, TAO ZHANG, JIANGUANG XU

**Affiliations:** Department of Orthopaedics, The Sixth People’s Hospital of Shanghai, Shanghai Jiaotong University, Shanghai 200233, P.R. China

**Keywords:** spine surgery, thoracolumbar fracture, internal fixation, computer-assisted surgery, bone transplantation

## Abstract

The aim of this study was to investigate the method of posterior thoracolumbar vertebral pedicle screw reduction and fixation combined with vertebral bone implantation via the affected vertebral body under navigational aid for the treatment of thoracolumbar fractures. The efficacy of the procedure was also measured. Between June 2005 and March 2011, posterior thoracolumbar vertebral pedicle screw reduction and fixation plus artificial bone implantation via the affected vertebral pedicle under navigational aid was used to treat 30 patients with thoracolumbar fractures, including 18 males and 12 females, ranging in age from 21 to 57 years. Compared with the values prior to surgery, intraspinal occupation, vertebral height ratio and Cobb angle at the follow-up were significantly improved. At the long-term follow-up, the postoperative Cobb angle loss was <1° and the anterior vertebral body height loss was <2 mm. Posterior thoracolumbar vertebral pedicle screw reduction and fixation combined with vertebral bone implantation via the affected vertebral body under navigational aid may increase the accuracy and safety of surgery, and it is an ideal method of internal implantation. Bone implantation via the affected vertebral body may increase vertebral stability.

## Introduction

Thoracolumbar fracture is most common trauma in spine surgery (1–3) and is usually a high energy trauma caused by a traffic accident or fall. With the rapid development of the economy and the popularization of cars, the thoracolumbar fracture incidence rate is increasing year by year (2–4). The aim of recovering vertebral height, maintaining vertebral stability by internal fixation and enabling ambulation as early as possible has become established among physicians in spine surgery. For the conventional posterior reduction and fixation of thoracolumbar fractures, long-term follow-up shows cases of vertebral height loss of the affected vertebral body (5–7). In addition, computer technology has improved rapidly in recent years. Computer-assisted surgery (CAS) was first applied in spine surgery in the 1990s (1) and is a novel guidance mode of implant placement. CAS is an assistive technology based on modern computer technology, stereo positioning techniques and medical imaging technology that is used to guide surgeons in precise surgical planning and surgery. The principle of a CAS system is similar to that of a global positioning system; it assembles a three-dimensional coordinate system of the intraoperative anatomical structure and a three-dimensional coordinate system of navigation images. Modern spinal surgery navigation and positioning systems mainly use infrared technology to identify anatomical structures of the patients and the mutual spatial relationship of the surgical implant with the surgical apparatus, and the computer provides virtual images of the internal fixation *in vivo* in multiple directions. Therefore, CAS may aid the surgeon in mastering the accurate positioning of the internal fixation *in vivo* in real-time (8) and create a radiation-free, multidimensional and virtual surgical environment during surgery (9). Although there are certain disputes, the use of CAS in spinal surgery, particularly in the pedicle screw transplantation process, is safer, more accurate and is accepted by an increasing number of spinal surgeons. Between June 2005 and March 2011, surgeons at The Sixth People’s Hospital of Shanghai (Shanghai, China) used CAS to conduct the reduction and fixation of pedicle screws and conduct artificial vertebral bone transplantation of 30 patients with thoracolumbar fractures via the affected vertebral pedicle and obtained a satisfactory efficacy.

## Materials and methods

### General data

In this study, there were a total of 30 cases, including 18 males and 12 females, and their ages ranged from 21 to 57 years (mean, 35.5 years). Among them, there were 17 cases of falling trauma, 9 cases of trauma caused by traffic accident and 4 cases of crashing traumas (high-energy injury). Of the fracture sites, 3 cases occurred in T11, 11 cases occurred in T12, 14 cases occurred in L1 and 2 cases occurred in L2. According to the Frankel method (10) of neurological dysfunction classification, 2 cases were classified as grade A, 3 cases were classified as grade B, 3 cases were classified as grade C, 7 cases were classified as grade D and 15 cases were classified as grade E. Fracture severity: intraspinal occupation was 5–70% (mean, 37.5%), vertebral height compression was 40–70% (mean, 54.5%) and Cobb angle was 15.5–41.5° (mean, 29.5°). The navigation equipment provided by Stryker Company (Kalamazoo, MI, USA) and used during surgery included a space-positioning device, an image workstation, a patient tracer and a surgical operation guide. This study was conducted in accordance with the declaration of Helsinki and with approval from the Ethics Committee of the Sixth People’s Hospital of Shanghai, Shanghai Jiaotong University. Written informed consent was obtained from all participants.

### Preoperative preparation

For all patients, conventional X-ray photography was conducted at the frontal and lateral positions, and MRI examination was conducted to assess the spinal cord injury. In addition, CT scanning was conducted for reconstruction. The CT scanning thickness was 1 mm and scanning was continuous. CT plain scanning of the vertebral body was 0.625–1.25 mm. CT scanning images included the vertebral body, spinal process and transverse process. In general, the images contained two adjacent upper and lower vertebral bodies. The CT scanning image of a single-segment vertebral fracture contained five vertebral bodies. CT scanning data were stored via compact disc. Prior to surgery, CT data in the compact disc were input into the computer navigational system for preoperative design. During registration, individual vertebral bodies were registered separately. The reference points should not be selected on the same plane. In our study, the superior margin of the spinous process was a required point, with two points selected on both sides of the spinous process. The reference points were not on the same plane. The upper spinous process was selected and the other four points were distributed at the two sides of the spinous process (two points at each side). In general, articular and transverse processes were selected as reference points. Vertebral compression, spinal bone block occupation, fracture displacement and deformity changes in the vertebral pedicle of the affected vertebral body were observed, and the insertion point, track and length of the screw channel were measured.

### Surgical techniques

**Figure 1.**
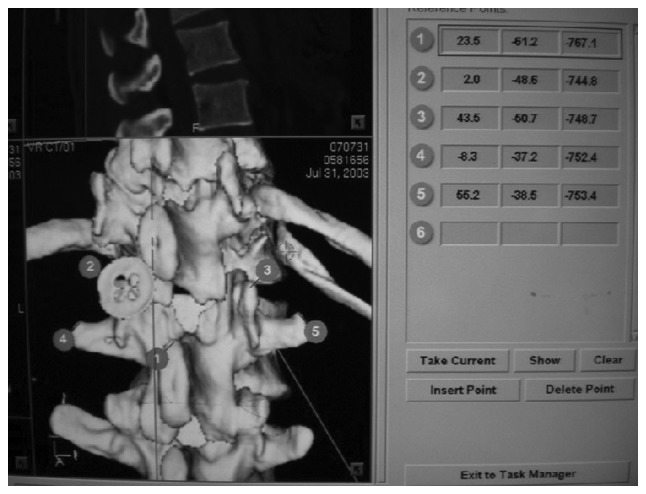
Reference points selected for preoperative registration.

**Figure 2.**
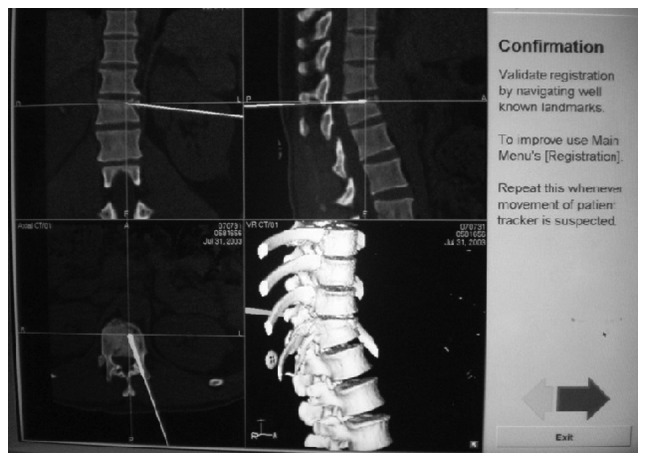
Intraoperative three-dimensional navigation chart.

**Figure 3.**
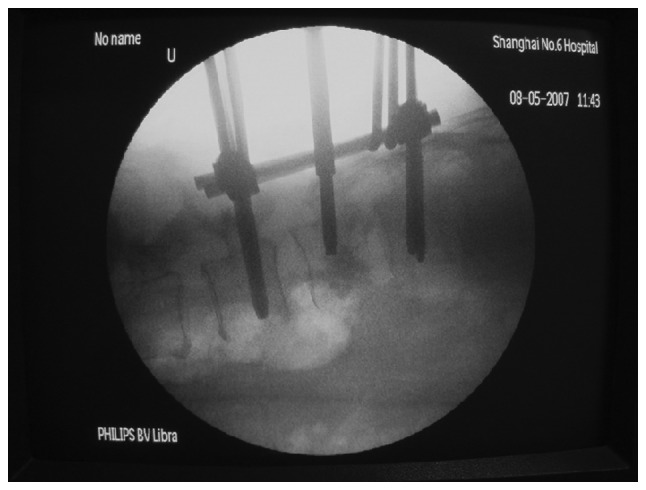
Intraoperative bone implant status examined by C-arm X-ray.

**Figure 4.**
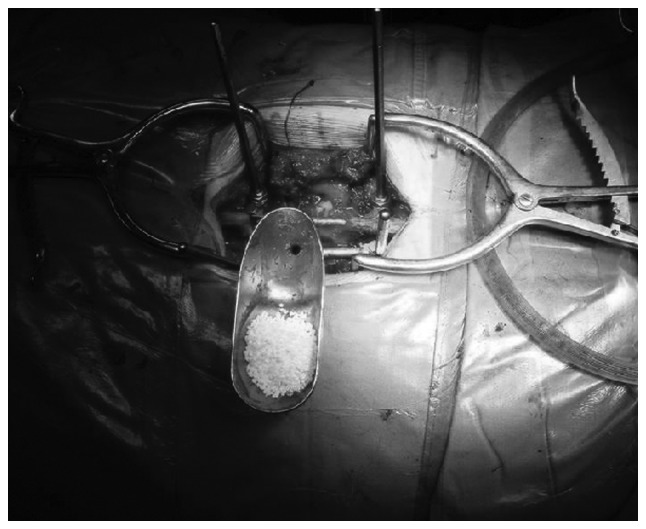
Intraoperative bone transplantation.

**Figure 5.**
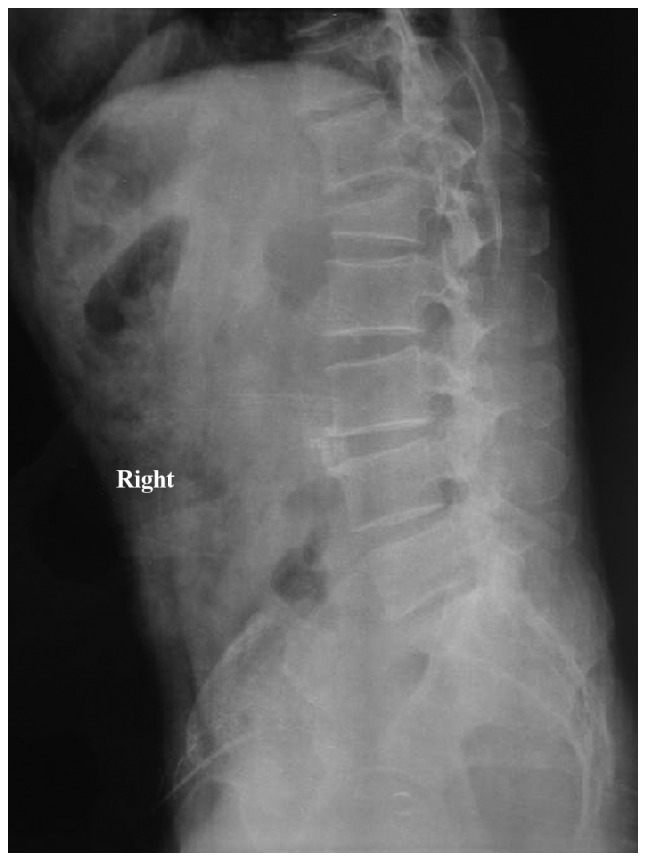
Preoperative X-ray film of a 45-year-old male patient showed a fracture at L1. The height of L1 was compressed to a half of that of a normal vertebral body.

**Figure 6.**
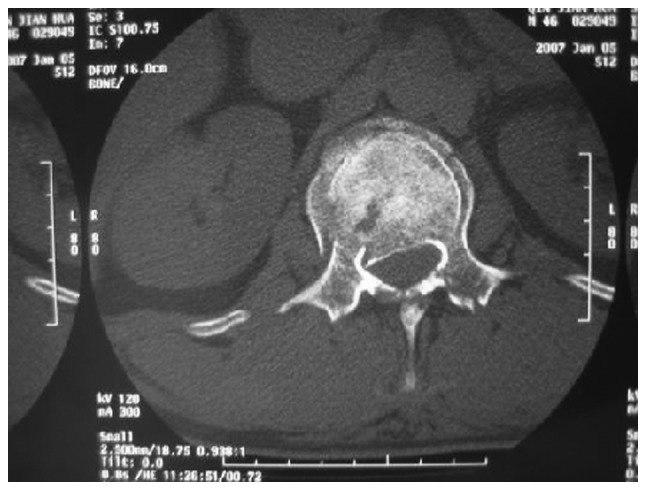
Preoperative CT scanning image of a 45-year-old male.

**Figure 7.**
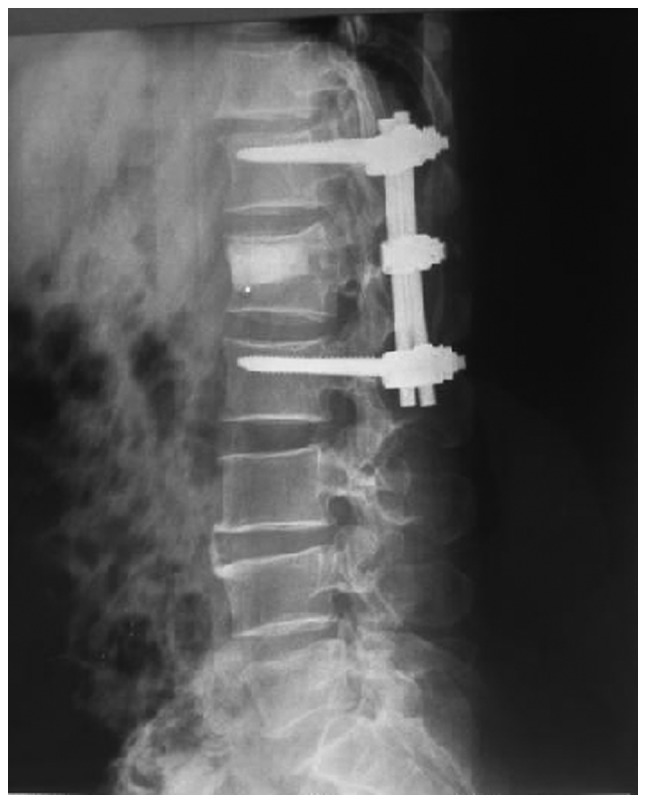
Postoperative X-ray film of lumbar vertebra of a 45-year-old male.

**Figure 8.**
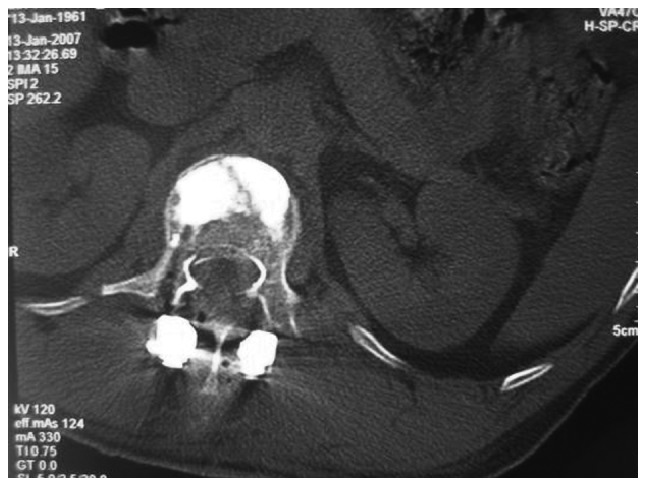
Postoperative CT scanning image of a 45-year-old male.

**Figure 9.**
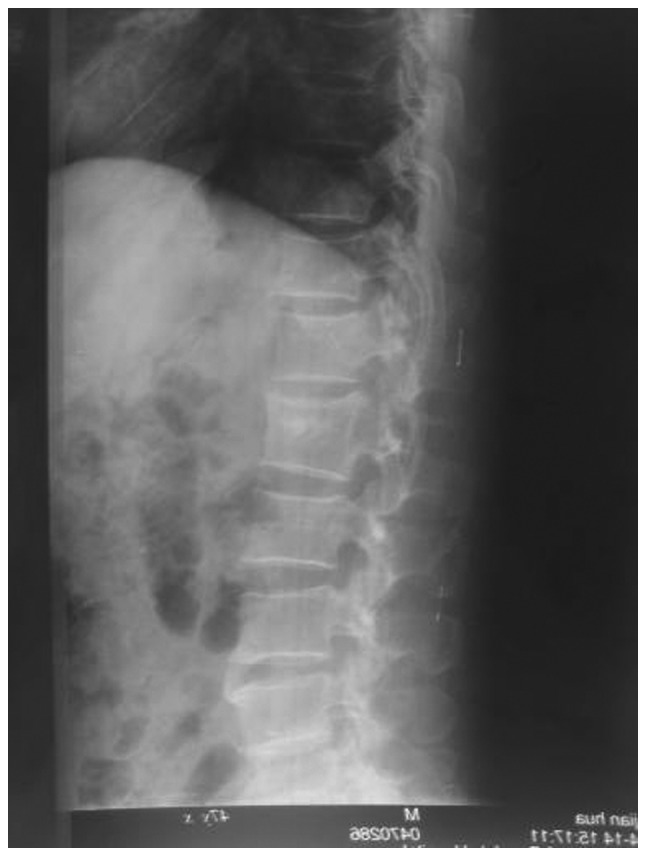
X-ray film following removal of the internal fixation at one year after surgery of a 45-year-old male.

**Figure 10.**
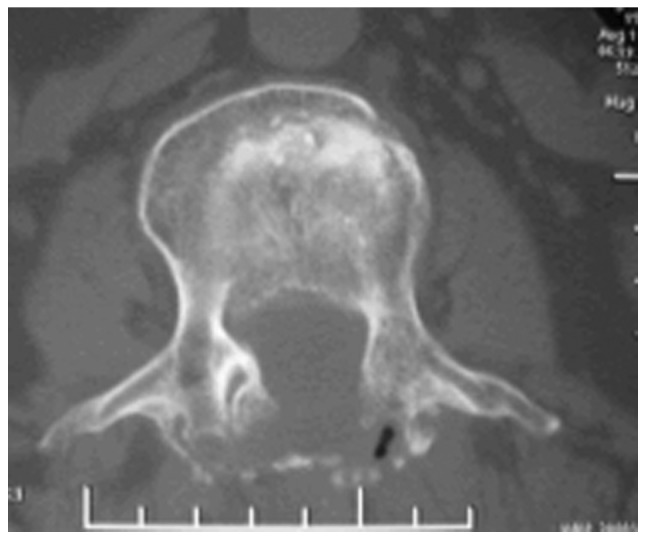
CT scanning image following removal of the internal fixation at one year after surgery of a 45-year-old male.

General anesthesia was conducted and patients were in the prone position. The posterior median incision was cut open to expose the affected vertebral body, articular process joint and the transverse process at the upper and lower segments. A tracer was installed on the spinous process where pedicle screws would be implanted. It was necessary to ensure the tracer was fixed solidly. The position sensor was adjusted, and the radio calibrator (a necessary instrument for a navigation surgery; Stryker Company, USA) was registered and calibrated. According to the five reference points designated prior to surgery, control matching was conducted. It was essential that the reference point on the upper spinous process was preserved. Two reference points (one reference point with the largest matching error at each side of the spinous process) were deleted. For the remaining three reference points, the error range was automatically calculated by the navigational system. If the error range was <1.0 mm, it was acceptable (Fig. 1) and it was feasible to operate under the guidance of the navigation model. Each time, intraoperative surgical tools were registered and calibrated in order to be displayed on-screen following infrared receiver reconnaissance (Fig. 2). Following the implantation of two groups of four vertebral pedicle screws, C-arm X-ray examination was conducted to confirm the position of the vertebral pedicle. For the affected vertebral body, a small hole was created at the vertebral pedicle by cutting under the guidance of the navigation model and the bone-opening instrument was placed into the vertebral body along the vertebral pedicle to the empty cavity of the fracture. The screw channel was closed with bone wax temporarily to reduce vertebral hemorrhage. If the fracture was accompanied by neurological dysfunction, full decompressive laminectomy and spinal nerve exploration were conducted. Following pedicle screw reduction, C-arm X-ray examination was conducted to confirm the reduction status of the affected vertebral body. Following satisfactory reduction, all screws were tightened (Fig. 3). A bone implant funnel was inserted from one side of the affected vertebral pedicle screw channel and 5 g particulate artificial bone was implanted into the funnel and pressurized into the vertebral body with the filling rod. Subsequently, the screw channel opening was closed with bone wax. For the contralateral screw channel, 5 g particulate artificial bone was implanted in the same manner (Fig. 4). The wounds were closed layer by layer. Following surgery, negative pressure drainage was conducted for 48 h (Figs. 5–10).

### Postoperative treatment

Following surgery, the patients lay in bed for 2–4 weeks, then started to ambulate under waist protection. For all cases, postoperative plain CT scanning was conducted to observe the accuracy of the vertebral pedicle screw implantation. According to the possibility of the screw penetrating the vertebral pedicle and penetration extent, the pedicle screw was classified into 4 grades. If the pedicle screw was within the vertebral pedicle, it was classified as grade 0; if the pedicle screw was involved in the vertebral pedicle cortex, it was classified as grade I; if the depth of the pedicle screw penetrating the cortex was <2 mm, it was classified as grade II; and if the depth was >2 mm, it was classified as grade III. In addition, the bone implantation status of the affected vertebral body and the intravertebral fracture block occupation and reduction status were observed.

### Efficacy evaluation and observation indices

Following surgery, X-ray re-examination was conducted regularly to measure the vertebral height and Cobb angle. With the affected vertebral body positioned in the center, frontal and lateral X-ray plain films were photographed at 3, 6, 9 and 12 months after surgery and at 3 months following removal of the internal fixation. On the lateral X-ray plain film, two straight lines were drawn along the upper endplate of the upper adjacent vertebral body of the affected vertebral body and the lower endplate of the lower adjacent vertebral body, and the crossing angle of the two lines was the Cobb angle of the affected vertebral body.

At 12 months following the surgery, plain CT scanning was conducted for re-examination to measure the intraspinal occupation and artificial bone replacement status. Intraspinal occupation and vertebral height ratio following surgery and at the final follow-up were compared with those prior to surgery. According to the lower back pain efficacy assessment standard of the Japanese Orthapaedic Association (JOA) (7), JOA scores prior to surgery and at the final follow-up were assessed. According to the visual analog score system (VAS) evaluation standard, VAS scores of pain prior to surgery and at the final follow-up were assessed.

### Statistical analysis

SPSS 13.0 statistical software (SPSS, Inc., Chicago, IL, USA) was used and measurement data, including the vertebral height, Cobb angle and intraspinal occupation, were expressed as the mean ± standard deviation. Repeated measures analysis of variance was conducted for comparisons of various indicators prior to and following surgery and at the final follow-up, and least significant difference t-test was used for intra-group pairwise comparison. P<0.05 was considered to indicate a statistically significant result.

## Results

### Surgical duration and bleeding volume

Surgical durations ranged from 90 to 130 min and the mean duration was 105.2±26.3 min. The bleeding volumes ranged from 150 to 800 ml and the mean bleeding volume was 279.7±173.7 ml. Intraoperative C-arm X-ray examination was conducted once or twice.

Among 120 vertebral pedicle screws, 110 pieces were classified as grade 0, 8 pieces were classified as grade I and 2 pieces were classified as grade II. Successful screw rate was 98.3% and no piece was classified as grade III. Follow-up durations ranged from 12 to 36 months (mean, 18 months). At 3, 6, 12 and 24 months after surgery, follow-ups were conducted. At each follow-up, X-ray examination was conducted. Thoracolumbar fracture was more common in young adults. At >1 year after surgery, the internal fixation was removed in order to prevent vertebral pedicle screw breakage and plain CT scanning was conducted. According to postoperative Frankel classification, 2 cases were grade A, 2 cases were grade B, 1 case was grade C, 7 cases were grade D and 18 cases were grade E. Postoperative intraspinal occupations ranged from 0 to 20% (mean, 14.3%) and vertebral heights ranged from 80 to 100% (mean, 91.3%). Cobb angles ranged from 1.3 to 9.1° (mean, 4.9°). At the final follow-up, intraspinal occupations ranged from 0 to 25% (mean, 14.3%), vertebral heights ranged from 80 to 100% (mean, 90.7%) and Cobb angles ranged from 1.6 to 8.6° (mean, 5.1°). Following long-term follow-up, postoperative Cobb angle loss was <1°, vertebral height loss was <2 mm and there was no screw breakage or internal fixation loosening (Table I). Typical case images are shown in Figs. 5–10.

### Surgical complications

Among the cases in the formation group (receiving artificial bone transplantation into the injured vertebral body), the weights of the individual artificial vertebral bone implants ranged from 10 to 25 g and the mean weight was 18.64 g. Among them, 2 cases presented anterior vertebral body leakage and the leakage was absorbed naturally over 3 months. No case presented intraspinal leakage. Following surgery, no neurological complication and no surgical complication in other vessels, nerves or organs occurred.

### JOA score results

The lower back pain efficacy assessment standard used was that prepared by the JOA (7) which is mainly used for the evaluation of postoperative efficacy in thoracolumbar vertebral diseases. This standard is brief, clear and widely applied in the clinic. A normal total score is 29 points; the higher the score, the greater the efficacy. The preoperative mean JOA score was 11.73 (11.73±2.94) and JOA score at the final follow-up was 27.53 (27.53±3.01). The VAS score is mainly evaluated according to the subjective pain sensation of patients (8). The score range is 0–10 points and if the subjective pain sensation is more severe, the score is higher. The mean preoperative VAS score was 6.83 (6.83±0.91) and the mean VAS score at the final follow-up score was 9.17 (9.17±0.27).

## Discussion

Lordosis and kyphosis are different types of postural disorders which cause a physiological curvature of the thoracolumbar spine. Thoracic vertebrae are fixed relatively due to rib support and lumbar vertebra are highly mobile. Such anatomical features easily cause thoracolumbar vertebral fracture in casew of trauma. In particular, vertebral fracture at T12-L1 is the most common and is usually accompanied by injuries to the cauda equina and other injuries. At present, unstable spinal cord injuries are mostly treated by surgery (11–14). The surgery restores the integrity and stability of the spinal anatomical structure as far as possible in order to create favorable conditions for recovery of neural function. Spinal anatomical reduction and bone fusion may effectively prevent malformation and reduce chronic disability to enable early exercise. If the fracture is accompanied by neurological dysfunction, decompressive laminectomy and spinal nerve exploration are conducted according to the disease conditions. If damage to the endorachis is visible, it is repaired as far as possible. However, this is only to create favorable conditions for neurological recovery and not all nerve injuries may be restored.

In cases of vertebral fracture, the lamina terminalis is damaged, the intervertebral disc is pushed into the vertebral body and the normal bone trabecula support system in the vertebral body is damaged. Although the vertebral height may be fully be restored, compressed bone trabeculae are not restorable, which results in a vertebral body with lack of bone integrity. It is difficult to form bone by hematoma organization and cartilaginification mechanisms, and it is only feasible to fill the empty defect (namely, a so-called ‘eggshell’ or ‘empty’ vertebral body) with fibrous tissues (15–17). On X-ray plain film, the manifestations may be normal. Occasionally, mild depression of the affected vertebral lamina terminalis may be visible. However, plain CT scanning shows an empty cavity on the affected vertebral body. According to the three-column spinal column theory by Denis (18), the vertebral body and the posterior longitudinal ligament respectively constitute the anterior and central columns of the spinal column. The stability and loading of the spinal column primarily depend on the anterior and central columns, which account for approximately 60% of the total load of the spinal column. The spinous process and vertebral lamina constitute the posterior column of the spinal column. Therefore, pedicle screw fixation and lamina posterolateral bone graft fusion during operation are not reliable for the stability maintenance of the integral spinal column, namely that strengthening the stability of the posterior column merely has no noticeable effect on the stability of the anterior and central columns. Plain CT scanning images of the fracture at the long-term follow-up show that the empty intravertebral cavity is always present (19,20). As vertebral stability is mainly involved in the anterior middle spine (accounting for ∼60%), performing posterolateral fusion during surgery is not reliable and is ineffective for the stabilization of the anterior middle spine. Bone transplantation of the affected vertebral body is controversial and not all vertebral fractures require bone transplantation (21,22). Bone transplantation of the affected vertebral body is recommended for thoracolumbar unstable fractures, including vertebral fractures with compression >1/3, vertebral burst fractures, fracture fragment intrusion into the spinal canal and cases of a larger empty cavity in the vertebral body following vertebral body reduction and in which long-term vertebral height loss is easily generated (23–26). The artificial bone transplanted into the vertebral body is used as filler to strengthen and support the affected vertebral body and thus reconstruct the stability of the anterior middle spine. Therefore, the artificial bone may effectively resist axial load to avoid the ‘eggshell’ effect and effectively prevent long-term vertebral height loss to reduce kyphosis deformity and to reduce the incidence rate of long-term complications (27). Artificial bone may induce bone growth and act as a bone support for bone creeping substitution. For the affected vertebral body, spinal stability may be fully restored only by autologous bone fusion. It is undesirable to conduct internal fixation without bone fusion.

Internal fixation technology has developed greatly, and simple anterior and posterior approach surgeries have certain advantages and shortcomings (28–30). We applied short-segment posterior fixation reduction combined with artificial bone transplantation via the affected vertebral pedicle to treat thoracolumbar fractures. This combines the advantages of the anterior and posterior approach surgeries and overcomes their shortcomings to conduct reduction, decompression and reconstruction of the injured spine in a one-step process. The bone implant funnel may be directly inserted into the anterior middle spine of the vertebral body via the affected vertebral pedicle. The filling rod for bone transplantation is used to directly place the artificial bone into the empty cavity and uniformly distribute it in the empty cavity of the anterior middle spine of the vertebral body. Its effect is to fully fill the intravertebral empty cavity following reduction, which improves the load-bearing capacity of the affected vertebral body and creates more reliable vertebral stability. Solid artificial bone has no toxicity and low leakage. In instances of leakage, solid artificial bone does not cause risks and may naturally degrade and be absorbed. In addition, its biological compatibility with tricalcium phosphate is good. Once the solid artificial bone has been fully degraded by body fluid degradation and cytophagy *in vivo*, it is absorbed. Following degradation, released calcium and phosphorus are able to directly participate in new bone mineralization or enter the calcium and phosphorus banks for further use.

There are three methods of image acquisition using navigational systems: CT navigation, X-ray navigation and intraoperative real-time three-dimensional imaging (31). The advantages of the CT navigational system are that it enables preoperative design and planning to be conducted and may be used for intraoperative 3D image guidance. The shortcomings of the CT navigational system include that it is necessary to acquire images prior to surgery and it is impossible to update images during surgery. The advantages of the X-ray navigational system include that it is not necessary to acquire images prior to surgery and it is possible to update images during surgery. Shortcomings include that it is impossible to conduct preoperative planning and the image definition is poor. In addition, no three-dimensional image is provided for reference. Intraoperative three-dimensional imaging combines the advantages of the other two methods and overcomes their shortcomings. The quality of the three-dimensional images obtained during the operation was restricted by the adopted machine and software. They were less clear than those obtained by the navigator before CT plain scanning. In particular, the three-dimensional reconstruction image is much worse. Due to the characteristics of the CT and X-ray navigational systems, a CT navigational system was used for all cases in this study. As the vertebral pedicle diameter of the thoracolumbar vertebral body is relatively large and the anatomic structure of the vertebral body has less variation, surgical navigation is relatively simple. However, as vertebral fracture situations differ according to the injury extent of the affected vertebral body, particularly in cases of fracture displacement of the affected vertebral pedicle and the loss of important anatomical landmarks, including the articular process joint and fracture displacement of the transverse process, the drilling difficulty and risk associated with the affected vertebral pedicle screw are markedly increased (32). During surgery, it is impossible to comprehensively and accurately master the actual situations of the affected vertebral body according to C-arm X-ray examination. The surgery conducted at this time has a certain lack of visibility and risk. The use of a CT navigational system may comprehensively and accurately indicate the extent of injury of the affected vertebral body, the involvement of the vertebral pedicle, articular process joint and transverse process in the fractures and the possibility of malformation. Therefore, it is feasible to prepare a detailed preoperative plan for the determination of screw channel length, placement site and screw channel track, which make bone implantation more accurate, effective and safe. During surgery, it is feasible to conduct real-time monitoring of the screw channel under the visual guide of a three-dimensional image and change the three-dimensional direction of the drilling equipment and drilling depth in a timely manner to accurately reach the bone transplantation site. Subsequently, the surgery is accurate and effective, the risk associated with the surgery is greatly reduced, and intraoperative accidents are reduced. In addition, the surgery duration is shortened.

The CT navigational system has clear advantages for bone transplantation, but also has shortcomings. The preoperative CT body position of a patient may be different from the intra-operative body position to a certain extent and the fracture situations may be slightly different. If the difference is great, navigation accuracy will be affected, requiring the operator to conduct a multiple-point registration of the anatomical structure of the patient to correct the difference and increase the real-time accuracy of this navigation mode (33,34). If necessary, C-arm X-ray examination is conducted for confirmation. Numerous physicians speculate that the use of navigational aids is likely to increase surgical duration, but that was not observed to be the case in the present study. The main difference between surgical navigation and routine surgery is that individual vertebral bodies must be registered individually. If the operator is skilled in surgery, registration only takes a few minutes and the number of C-arm X-ray examinations is reduced. Certain scholars consider that the relative degeneration of fracture patients is milder and the anatomical landmarks are clear and question whether it is necessary to apply a navigation system for a non-fractured vertebral body. We consider that since a navigational aid is used during surgery, it is feasible to apply the navigational system to non-fractured vertebral bodies. Since it is possible to conduct multi-point surgery, there are no risk or cost issues, although the registration time is increased.

In summary, the instantaneous tracking function of the navigational system enables the surgeon to monitor the surgical tool in real-time and accurately guide the arrival of the implant. It allows surgery to be visualized in multi-dimensions and real-time, making it an ideal implant guide. It is likely that navigational systems will be accepted by an increasing number of physicians. For physicians in spine surgery, vertebral pedicle screw implantation is a basic skill which is mastered expertly. At present, computer navigation technology plays only an auxiliary role and not a leading role. Navigational systems should not be excessively relied on, however, they should not be ignored. With the rapid development of computer technology, navigational aids will be used more widely. The treatment of posterior vertebral pedicle screw system fixation plus intravertebral bone transplantation via the vertebral pedicle for thoracolumbar fracture aids the restoration of normal spinal physiological structure and curvature. In addition, intravertebral bone transplantation with particulate artificial bone via the affected vertebral pedicle enables effective filling of the intravertebral bone defect cavity and strengthens the affected vertebral body to avoid postoperative vertebral height loss.

## Figures and Tables

**Table I.** Intraspinal occupation, vertebral height ratio and Cobb angle of 32 patients before and after surgery and at the final follow-up (mean ± SD, n=30).

Time	Intraspinal occupation (%)	Vertebral height (%)	Cobb angle (°)
Preoperative	37.5±32.5^a^	54.5±14.5^a^	29.5±12.5^a^
Postoperative	14.3±10.7	91.3±9.7	4.9±3.6
Final follow-up	14.3±10.7	90.7±9.3	5.1±3.5
F-value	4.939	3.386	5.892
P-value	0.000	0.000	0.000

a P<0.05, compared with that at the other time points.
